# Optical Genome Mapping as an Alternative to FISH-Based Cytogenetic Assessment in Chronic Lymphocytic Leukemia

**DOI:** 10.3390/cancers15041294

**Published:** 2023-02-17

**Authors:** Andriana Valkama, Sandra Vorimo, Timo A. Kumpula, Hannele Räsänen, Eeva-Riitta Savolainen, Katri Pylkäs, Tuomo Mantere

**Affiliations:** 1 Laboratory of Cancer Genetics and Tumor Biology, Translational Medicine Research Unit and Biocenter Oulu, University of Oulu, 90220 Oulu, Finland; 2 Northern Finland Laboratory Centre Nordlab, 90220 Oulu, Finland

**Keywords:** chronic lymphocytic leukemia, optical genome mapping, fluorescence in situ hybridization, chromosomal aberration, cytogenetics

## Abstract

**Simple Summary:**

In many countries, the standard-of-care cytogenetic analysis of chronic lymphocytic leukemia (CLL) is based on the fluorescence in situ hybridization (FISH) technique. This offers only a very targeted view of genomic alterations. Recent studies have demonstrated the potential of OGM as a cytogenetic tool for hematological malignancies. To confirm this for CLL, it is crucial to carefully evaluate the performance of OGM in detecting the routinely FISH-targeted aberrations in addition to its use as a genome-wide analysis method. We have evaluated the concordance of OGM and standard-of-care FISH in 18 samples from patients with CLL. Overall, the results were fully concordant between these two techniques. The genome-wide analysis revealed additional chromosomal aberrations in 78% of the samples and enabled the detection of complex karyotypes, which are undetectable by FISH. Based on our results, OGM could be used as a first-tier cytogenetic test for CLL.

**Abstract:**

The fluorescence in situ hybridization (FISH) technique plays an important role in the risk stratification and clinical management of patients with chronic lymphocytic leukemia (CLL). For genome-wide analysis, FISH needs to be complemented with other cytogenetic methods, including karyotyping and/or chromosomal microarrays. However, this is often not feasible in a diagnostic setup. Optical genome mapping (OGM) is a novel technique for high-resolution genome-wide detection of structural variants (SVs), and previous studies have indicated that OGM could serve as a generic cytogenetic tool for hematological malignancies. Herein, we report the results from our study evaluating the concordance of OGM and standard-of-care FISH in 18 CLL samples. The results were fully concordant between these two techniques in the blinded comparison. Using in silico dilution series, the lowest limit of detection with OGM was determined to range between 3 and 9% variant allele fractions. Genome-wide analysis by OGM revealed additional (>1 Mb) aberrations in 78% of the samples, including both unbalanced and balanced SVs. Importantly, OGM also enabled the detection of clinically relevant complex karyotypes, undetectable by FISH, in three samples. Overall, this study demonstrates the potential of OGM as a first-tier cytogenetic test for CLL and as a powerful tool for genome-wide SV analysis.

## 1. Introduction

Chronic lymphocytic leukemia (CLL) is a malignancy of mature clonal B cells and the most common form of leukemia in adults [1]. The clinical course of patients with CLL is extremely heterogeneous, as some may live for years without requiring treatment and have a normal life span while others undergo highly aggressive disease progression [2]. This heterogeneity is partly explained by the different genetic alterations that the cancerous cells harbor. Thus, for guiding the clinical management and prognosis of patients with CLL, sequence analysis of *TP53* and *IGVH* together with targeted cytogenetic testing is required [3,4]. The current standard-of-care (SOC) cytogenetic testing in CLL is often solely based on the fluorescence in situ hybridization (FISH) technique targeting selected genetic alterations of diagnostic and prognostic relevance. In Finland, among many other countries, CLL-FISH targets are limited to the deletions of 11q22.3 (*ATM*), 13q14.3 (*DLEU* region), and 17p13.1 (*TP53*) in addition to trisomy of chromosome 12 [5]. Patients with the loss of 17p13.1 and/or *TP53* mutations have the most adverse prognosis (very-high-risk group), followed by 11q deletions (high risk), and trisomy 12 or normal karyotype by FISH (intermediate risk). The most favorable prognosis is for patients with 13q14.3 deletion as a sole alteration (low risk) [6].

While the accurate detection of aberrations in these four loci is crucial, several studies have also demonstrated the importance of genome-wide profiling of structural variants (SVs) in CLL, especially to identify complex karyotypes that are associated with poor prognosis [7,8,9,10]. In addition, genome-wide analysis is essential for enabling the discovery of novel SVs and genes that may underlie the development and progression of CLL. Comprehensive analysis of SVs, however, requires complementary cytogenetic techniques, including chromosomal banding analysis (CBA) and chromosomal microarray (CMA)-based profiling of copy number variants (CNVs). As a downside of these techniques, the resolution of CBA in CLL is limited to ~10–20 Mb and requires modified cell culturing protocols to stimulate cell division [5]. The main limitation of CMA is that it does not detect balanced rearrangements, such as inversions and translocations, which are common in leukemias.

Optical genome mapping (OGM) is a novel non-sequencing-based technique for high-resolution genome-wide SV detection, and its use as an alternative to classical cytogenetic testing has been explored for various types of hematological malignancies [11,12,13,14,15,16,17]. Altogether, these studies have shown very high concordance between OGM and classical techniques, and for CLL specifically recent studies demonstrated the use of OGM as a powerful tool to assess genomic complexity [18,19]. OGM is based on the imaging of ultralong DNA molecules (average N50 of >240 kb) that are fluorescently labeled on a 6-mer single-stranded DNA motif that occurs on average 15 times per 100 kb in the human genome. Distinct label patterns that are generated during de novo genome assembly or extraction of divergent molecules from reference alignments allow the detection of various SV types (deletions, insertions, inversions, duplications, and translocations). Chromosomal aberrations that do not generate distinct label patterns, such as aneuploidies and terminal deletions, can be identified based on the molecule coverage depth information. Importantly, the OGM technique allows the cost-efficient generation of 300–500× genome-wide coverage, enabling the detection of low-level acquired SVs from cancer samples.

The aim of this study was to assess the capability and sensitivity of OGM, as a single test, to detect clinically relevant aberrations in the four loci routinely targeted by FISH in CLL. In addition, the use of OGM as a genome-wide SV analysis tool was evaluated.

## 2. Materials and Methods

### 2.1. Sample Selection

Mononuclear cell pellets, derived from bone marrow or blood, from 18 patients with CLL were obtained via The Finnish Hematology Registry and Clinical Biobank (FHRB Biobank, Helsinki, Finland: www.fhrb.fi). For each patient, the SOC FISH testing had been performed for 11q22.3 (*ATM*), 13q14.3 (*DLEU* region), 17p13.1 (*TP53*), and trisomy 12 (Vysis LSI p53/LSI ATM Probe set and LSI D13S319/LSI 13q34/CEP12 Multicolor Probe set). CBA or CMA analysis results were not available for these samples as they are not routinely performed for CLL. Samples were requested from the FHRB Biobank so that the studied cohort would include at least two positive samples for each of the four aberrations that are routinely tested with FISH.

### 2.2. OGM: DNA Extraction, Labeling, and Chip Run

All the OGM experiments were performed according to the manufacturer’s instructions using the Saphyr instrument and DLE-1 chemistry (Bionano Genomics Inc., San Diego, CA, USA). Ultrahigh-molecular-weight (UHMW) genomic DNA (gDNA) was extracted using Bionano Prep SP Blood and Cell DNA Isolation Kit according to the Bionano Prep SP Frozen Cell Pellet DNA Isolation Protocol (v2). Briefly, frozen cell pellets (each containing 10 M mononuclear cells) were thawed in a 37 °C water bath and suspended in DNA stabilizing buffer. From this suspension, 1.5 M cells were collected. Cells were lysed and digested with proteinase K, RNase A, and buffer LBB, and treated with PMSF (Sigma-Aldrich, St. Louis, MO, USA). The gDNA was precipitated with isopropanol and bound to a nanobind disk, then washed and eluted. The gDNA samples were mixed with HulaMixer (ThermoFisher Scientific, Waltham, MA, USA) for 1 h and equilibrated overnight at room temperature to homogenize the samples before quantification with Qubit Fluorometer 3.0 (Qubit BR dsDNA assay kit; ThermoFisher Scientific).

The extracted UHMW gDNA was labeled with Direct Label and Stain (DLS) technique according to manufacturer’s protocol (Bionano Prep DLS Labeling Kit; Bionano Genomics). For each sample, 750 ng of gDNA was labeled with DL-green fluorophores at 6 bp CTTAAG sequence motif using Direct Labeling Enzyme 1 (DLE-1). After proteinase K (Qiagen, Hilden, Germany) digestion, DL-green cleanup was performed with DLS membranes, and the backbone of the labeled gDNA was stained overnight at room temperature before Qubit Fluorometer quantification (Qubit HS dsDNA assay kit, ThermoFisher Scientific).

Following the quantification, the fluorescent-labeled UHMW gDNA samples were loaded on Saphyr chips (G2.3) and run on the Saphyr instrument. The amount of data to be collected was set to 1800 Gbp, and GRCh38/hg38 was used as the reference genome.

### 2.3. OGM Data Analysis

OGM data analysis was performed using the rare variant pipeline (RVP) included in Bionano Solve software (v3.7) and visualized in Bionano Access software (v1.7). The analysis was performed independently by two investigators, one blinded and the other nonblinded to the SOC FISH results. For each sample, the first analysis step focused on the four FISH-targeted loci using the recommended confidence scores for SV and CNV calling (insertion: 0, deletion: 0, inversion: 0.7, duplication: −1, intra-translocation: 0.05, inter-translocation: 0.05, copy number: 0.99, and aneuploidy: 0.95). For CNV calling algorithm, the recommended size cutoff of 500 kb was used. For SV calling, all the SVs present in the OGM control database (303 individuals) provided by Bionano Genomics were filtered out. In case a sample remained negative for aberrations in the FISH-targeted loci, an additional step of loosening the CNV tool and aneuploidy calling confidence values to ‘all’ and visually inspecting the regions was taken to detect putative low-level aberrations in these loci.

For the genome-wide analysis, the recommended confidence scores were used for both SV and CNV calling, and all the SVs present in the population control database were excluded. The analysis was focused on aberrations > 1 Mb in size unless they were overlapping with a known leukemia-associated gene (Table S1). For identifying acquired clonal copy neutral loss of heterozygosity (CN-LOH), the de novo assembly pipeline (v1.7) was used. For this purpose, the amount of data of each sample was downsampled to ~100× genome-wide coverage before running the computationally more demanding de novo assembly. For the CN-LOH calling, the recommended default settings and a size cutoff of 25 Mb was used, and the analysis was focused only on events that involved telomeric chromosomal regions [20].

### 2.4. Determining Lowest Limits of Detection by In Silico Dilution Series

To determine the lowest limits of detection with OGM, an analysis based on in silico dilution series for representative aberrations was performed. This included ~1 Mb sized deletion of 13q14 region (Chr13:49,971,221–51,052,363), ~17 Mb sized deletion of 11q region (Chr11:98,387,863–115,852,927), loss of 17p whole-arm, and trisomy 12. In addition, a balanced translocation t(14;18)(q32;q21) was included in this analysis outside the FISH-panel-targeted aberrations. The dilutions were performed within the Bionano Access software by combining imaged molecule data from different CLL samples harboring different aberrations and running the RVP on the combined molecule data (Table S2). For uniformity, only data from samples with map rates > 90% and similar run metrics were combined. Stepwise dilutions were performed by incrementally increasing the proportion of molecules derived from the wild-type sample until the targeted aberration was undetectable with the RVP analysis (as presented in Figure S1 for trisomy 12).

## 3. Results

### 3.1. Technical Metrics and Overall Number of SVs and CNVs

Sample preparations and runs were successful for all the 18 samples, and a minimum of 1800 Gbp of data was collected for each sample. This resulted in an average of 501-fold genome-wide coverage per sample (min: 420, max: 541). Map rates were above the recommended 70% for all the samples (average: 89%), although three of the samples had label densities slightly below the recommended range of 14–17 labels per 100 kb (min: 13.1 and max: 16.45 for the whole cohort). Each of the samples had a molecule length N50 above 258 kb (average: 298 kb) (Table S3). The median number of SV calls without size cutoffs per sample was 28 (min: 7, max: 79) when applying the recommended confidence scores and SV population frequencies set to 0%. The median number of CNV calls (segments > 500 kb) and aneuploidies combined was 3 (min: 0, max: 32) (Table S4).

### 3.2. OGM Results Compared to FISH

**Figure 1:**
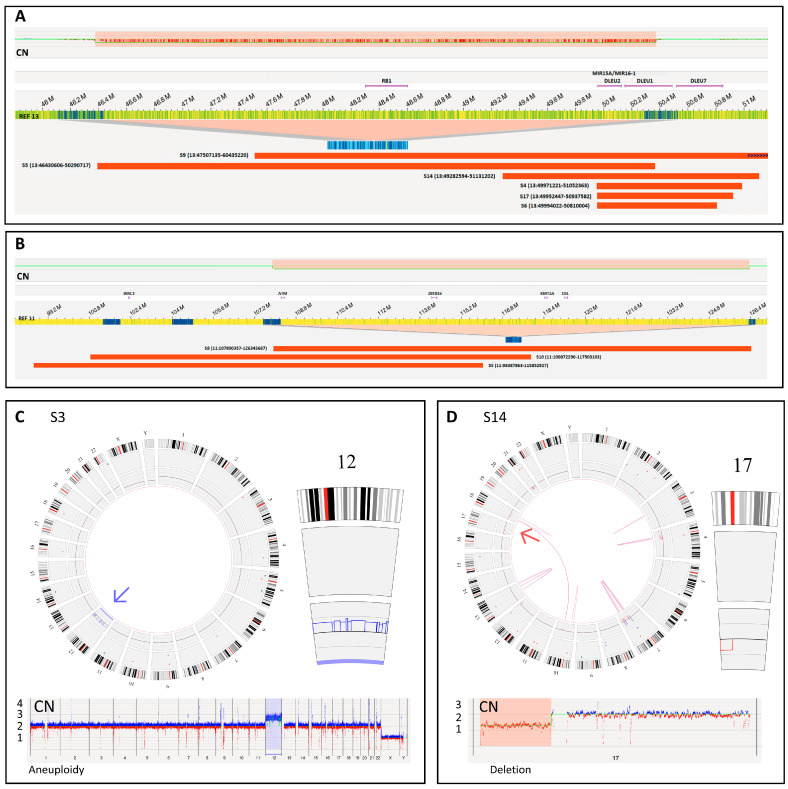
Visualization of aberrations detected by OGM in SOC FISH-targeted loci. (**A**) Genome map view showing the sizes and locations of the six different 13q14.3 (*DLEU* region) deletions, of which two also encompassed the *RB1* tumor suppressor gene. (**B**) Genome map view of the three different 11q22.3 (*ATM*) region deletions also involving other leukemia genes. (**C**) Circos plot view and copy number (CN) profile view of a sample with trisomy 12. (**D**) Circos plot view and CN profile of a sample with a deletion of the short arm of chromosome 17 (*TP53* deletion).

The analyzed 18 samples from patients with CLL harbored a total of 16 chromosomal aberrations that were previously detected by SOC FISH analysis, with the number of positive cells varying between 13 and 96%. Importantly, all 16 aberrations were identified both by the blinded and nonblinded OGM analysis using the standard settings without any adjustments to the analysis parameters (Table 1, Figure 1A–D). In total, these aberrations included three 11q22.3 deletions, four cases of trisomy 12, a monosomy 12, six 13q14.3 deletions, and two 17p13.1 deletions. For 8 of these 16 aberrations, OGM could also provide additional information either regarding other closely located leukemia genes or larger chromosomal rearrangements accompanying the aberration. For the 11q22.3 and 13q14.3 deletions, compared to FISH, OGM allowed us to determine more precise sizes and locations. All six 13q14.3 deletions (sizes: 0.8–3.9 Mb) involved the *DLEU1* and *DLEU2* genes, and in two samples the deletion encompassed the tumor suppressor gene *RB1* (Figure 1A). OGM also revealed that in these two samples, the 13q14.3 region was involved in larger chromosomal rearrangements, including translocation t(13;21)(q14.3;q22.3) and intrachromosomal rearrangements of chromosome 13 (Figure S2). All three 11q22.3 deletions involving *ATM* (sizes: 16, 17, and 18 Mb) also encompassed at least two of the other nearby leukemia-associated genes (*BIRC3, ZBTB16, KMT2A*, or *CBL*) (Figure 1B). In addition, for the two samples with 17p13.1 deletion (*TP53*), OGM analysis showed that the complete short arm of chromosome 17 was lost (Figure 1D). Regarding trisomy 12, FISH and OGM were concordant, and no additional information was gained with OGM. However, for sample S7, the FISH results were initially interpreted as monosomy of chromosome 12, but based on OGM analysis, a more complex CNV profile and structural rearrangements were evident (Figure S3).

### 3.3. Lowest Limits of Detection with OGM

Series of in silico dilutions were performed to estimate the lowest limit of detection for the FISH-targeted aberrations. Sizes of the aberrations included in the analysis ranged from a 1 Mb deletion (13q14) to a full trisomy of chromosome 12, and the detection required the use of both SV and CNV calling (including aneuploidy calling) algorithms, depending on the aberration type. The lowest limit of detection was below 10% variant allele fraction (VAF) for each of the tested aberrations (Table S2). Trisomy 12 was detectable down to 3% VAF when applying less stringent aneuploidy call confidence scores (Figure S1). Of note, allowing a less stringent confidence score did not yield any additional aneuploidy calls in our study cohort when tested. Based on the SV algorithm calls, a representative 17 Mb sized 11q deletion was detectable down to 5% VAF. For the 1 Mb sized 13q14 deletion, both the SV and CNV tool were able to detect the aberration at ~9% VAF. The whole-arm deletion of chromosome 17 was detectable down to 8.5% VAF when allowing less stringent confidence scores for CNV calling. Also here, allowing less stringent confidence CNV calls did not produce an excess of terminal deletions, or other larger (>5 Mb) CNV calls. Outside the FISH-targeted loci, t(14;18)(q32;q21) (the *IGH::BCL2* rearrangement) was chosen to represent a balanced translocation, and based on the performed dilution series OGM could detect this aberration down to 4% VAF (Table S2).

### 3.4. Genome-Wide Analysis

For each sample, a genome-wide analysis was performed to identify > 1 Mb aberrations (including telomeric CN-LOH regions) and also smaller SVs overlapping with known leukemia genes (Table S1). In addition to the fully concordant results between SOC FISH and OGM, large (>1 Mb) aberrations beyond the FISH-targeted loci were identified in 78% (14/18) of the samples (Table S5). Overall, several aberrations that have previously been described in CLL were identified in the cohort. These included a concomitant loss of 8p and gain of 8q [21], gains of the full short arm of chromosome 2 [22,23], loss of the full short arm of chromosome 18 [24], 15q deletion involving *MGA* [25,26], and two balanced translocations involving *BCL2*: t(14;18)(q32.33;q21.33) and t(2;18)(p11.2;21.33) (e.g., the *IGH::BCL2* and *IGK::BCL2* rearrangements, respectively) [27,28]. Importantly, the genome-wide analysis allowed us to identify three samples with complex karyotypes, all harboring five or more cytogenetic aberrations (Figure S3). The complex karyotypes were identified in two samples with the loss of *TP53* and in one of the samples with the loss of *ATM*. Interestingly, sample S7 with *TP53* loss displayed chromothripsis of chromosomes 3, 8, and 12, coupled with chromoplexy between chromosomes 8, 12, and 20, a phenomenon that has also previously been described in CLL [29].

The latest iteration of the Bionano’s de novo assembly pipeline (v1.7) allows the identification of constitutional CN-LOH regions but can also be used for CN-LOH calling in cancer samples with high cancer cell content. Overall, CN-LOH events involving telomeric regions were identified in four samples (one in a sample with complex karyotype and three in samples with simple aberrations) (Table S5). For sample S7, with a complex karyotype, a CN-LOH region covering most of the q-arm of chromosome 9 was identified. Sample S4, with a 1 Mb sized homozygous deletion including the 13q14.3 region, harbored CN-LOH covering almost the complete chromosome 13, in line with the homozygosity of the 13q14.3 deletion. Another CN-LOH region of interest was identified in sample S5, showing CN-LOH of Xq25–qter, involving four genes (*STAG2, BCORL1, PHF6,* and *BRCC3*) that have previously been implicated in different types of leukemias (Table S5).

In addition to the FISH-positive samples, the studied cohort included five samples that had remained negative in the SOC FISH analysis. Curiously, based on the OGM analysis, one of the samples harbored a ~16 kb sized insertion in the 3′ end of *ATM*, but whether this alteration actually disrupts *ATM* still requires further follow-up. Apart from this, these five samples were negative for aberrations in the four FISH-targeted loci. Importantly, OGM identified large cytogenetic alterations in four of these five FISH-negative samples. Sample S11, carrying the *ATM* insertion, harbored a previously unreported balanced translocation t(1;1)(q25.3;q41) with one of the breakpoints overlapping with *APOBEC4*, a member of the AID/APOBEC family of cytidine deaminases. Sample S12 showed a gain of 2p and a loss of 18p, both previously reported alterations in CLL [23,24]. Sample S15 carried an unbalanced translocation t(X;10)(26.2;26.3) resulting in a gain of Xq26.2–qter, and in addition small duplications and inversions overlapping the *MYC* proto-oncogene, which is known to undergo rearrangements in CLL transformation to Richter [30]. As a sole aberration, sample S16 harbored a previously unreported low-VAF balanced translocation t(2;8)(q37.1;q13.1), which could lead to a fusion of *SP110* and *COPS5* genes (Figure S4). Regarding putative novel gene fusions, another balanced low-VAF translocation t(Y;15)(q11.221;q21.2), potentially leading to a previously uncharacterized gene fusion between *GABPB1* and *UTY*, was identified in sample S18 with trisomy 12 (Figure S4).

## 4. Discussion

FISH has been the SOC cytogenetic technique for CLL since the early 2000s [6], guiding the risk stratification and clinical management of patients with this malignancy. However, several studies have also highlighted the prognostic importance of complex karyotypes, independent of the *TP53* and *IGHV* mutational status [8,31]. In addition, various individual genetic aberrations beyond the current SOC FISH panel may be of clinical relevance [26,28,32]. Regardless of these recognized additional genomic aberrations, genome-wide cytogenetic analysis using CBA and/or CMA is generally not performed for CLL. This is partly because combining multiple cytogenetic tests is very time-consuming and expensive, thus often not feasible in a diagnostic setup. CBA also suffers from poor resolution, a limitation which can be partly overcome with CMA-based CNV detection. However, CMA is unable to detect balanced structural variations, which is a major limitation, especially when analysing leukemia genomes. Recent studies have demonstrated the feasibility of OGM as a generic cytogenetic tool for hematological malignances [11,12,13,14,15,16,17,18], but to confirm this for CLL it is crucial to carefully evaluate the performance of OGM in detecting the most important routinely tested aberrations in this leukemia type in addition to its use as a high-resolution genome-wide analysis method.

In this study, based on 18 samples from patients with CLL, the results of the SOC FISH and OGM techniques were 100% concordant. It is also noted that the investigator performing the OGM analysis was blinded to the SOC FISH results, and the aberrations were identified using standard RVP analysis without modifications to the analysis parameters. Although the sample size of this study is limited, the results are well in line with previous investigations that have consistently reported > 90% concordance between OGM and standard cytogenetic techniques (FISH, CBA, and/or CMA) in various leukemia types [11,12,13,14,15,16,17,18]. For CLL specifically, two recent studies utilizing OGM demonstrated similarly high detection rates as our study (100% and 96%, respectively) for the SOC FISH-identified aberrations [14,18]. Importantly, OGM also provided more exact sizes and locations of the identified aberrations when compared to FISH. For example, this allowed us to directly determine whether the *RB1* tumor suppressor gene is included in the 13q deletion region or whether *BIRC3* is deleted in the case of del(11q). This is relevant as the disruption of these genes may be related to adverse prognosis in CLL [33,34].

All the FISH-targeted aberrations were detected by OGM in this study, but the most likely reason for missing an aberration in these loci is a low fraction of positive cells in a given sample. In our study, the number of positive cells varied between 13 and 96% in the FISH analyses. However, it is noted that the source material used for FISH and OGM differed in most cases (bone marrow versus blood, Table 1), which could partly explain some of the discrepancies observed in the levels of the detected aberrations with these two techniques in our study. All the analyzed samples were requested from the biobank without selection for the levels of positive cells. However, we estimated the lowest limits of detection by performing an in silico dilution series for each of the four aberrations. We determined that the FISH-targeted aberrations ranging from 1 Mb deletion (13q) to a full trisomy of chromosome 12 were detectable below 10% VAFs (range: 3–9%, depending on the aberration). Overall, our results regarding the lowest limits of detection are in line with previous studies, indicating that OGM can detect all types of clinically relevant cytogenetic aberrations in leukemia with the lowest limit of detection at ~10% VAFs [15]. Somewhat surprisingly, the molecule-depth-based aneuploidy calling was the most sensitive of these and was able to detect the trisomy 12 down to 3% VAF. These lowest limits of detection are similar, albeit slightly higher, when compared to the sensitivity of conventional diagnostic FISH analysis, which is somewhat probe-specific but commonly considered to range within 5–10% of positive cells [35]. In this study, the in silico dilution series demonstrated that for low-level aberrations requiring the CNV calling algorithm (17p arm deletion and trisomy 12), it is necessary to use less stringent confidence scores. Importantly, in our datasets this did not come with a price of additional potentially false-positive calls that could jeopardize the feasibility of a genome-wide analysis or make it difficult to distinguish a true aberration from noise. Thus, for diagnostic CLL samples, it would be recommended to take an additional analysis step to check for low-level 17p losses and trisomy 12 using less stringent analysis confidence scores. For future studies, it would also be important to evaluate the lowest limits of detection in samples with various, carefully quantified, low-level alterations.

Novel genes and aberrations are constantly being discovered and linked with biological and clinical features of hematological malignancies, including CLL [29,36]. Therefore, it is likely that a genome-wide analysis will become increasingly important for diagnostic CLL samples as well. In addition to the reliable and sensitive detection of the FISH-targeted aberrations, genome-wide OGM analysis detected additional (>1 Mb) SVs in the majority (78%) of the samples. These included multiple chromosomal aberrations that have previously been reported in CLL. Some of these have been proposed to be of clinical relevance, such as the IGH rearrangements (t(14;18)(q32.33;q21.33), t(2;18)(p11.2;21.33)) and the gain of 2p involving *MYCN* [22,28]. The genome-wide analysis also allowed us to identify three complex karyotypes. This is highly important as recent studies have suggested that complex karyotypes have prognostic significance and are associated with shorter survival and advanced disease and may be relevant for treatment decision making in CLL [8,9,10,37]. The detection of these complex karyotypes is not possible with FISH analysis. The capability to detect chromothripsis and chromoplexy is also an important feature of OGM as these complex rearrangements may play an important role in CLL pathogenesis [29]. In addition, OGM enabled the detection of CN-LOH regions, which may be important for the pathogenesis of CLL [38]. CN-LOH cannot be detected with FISH, while with OGM the detection of CN-LOH should, in principle, work similarly to SNP-based CMA. However, to our knowledge, there are no systematic studies on CN-LOH detection with OGM. With current analysis tools, CN-LOH detection is possible only for samples with constitutional CN-LOH or cancer samples with high cancer cell content in the case of acquired clonal CN-LOH.

We identified several novel SVs in the analyzed cohort. In particular, the previously unreported balanced translocations and the small insertion in *ATM* require further follow-up. Overall, it is possible that smaller SVs present a largely unexplored class of SVs that are currently missed by standard techniques in CLL. The novel balanced translocations (t(2;8)(q37.1;q13.1) and t(Y;15)(q11.221;q21.2)) could potentially lead to novel gene fusions (*SP110::COPS5* and *GABPB1::UTY*, respectively). The t(2;8)(q37.1;q13.1) translocation is particularly interesting as it was identified in an SOC FISH-negative sample that was also negative for all other aberrations in the genome-wide OGM analysis. In addition, recurrent deletions of chromosomal region 2q37.1 involving *SP110* have been previously reported in CLL [29]. These alterations certainly require further follow-up, but their identification already highlights the strength of the OGM technique in directly identifying genes within the breakpoints of novel translocations and identifying smaller SVs. For the novel SVs, it would also be important to know whether any of these are recurrent events in the given leukemia type. Leukemia sample cohorts analyzed with OGM from individual centers are still relatively small, and it would be highly important to establish collaborations and pool OGM datasets to enhance the identification of recurrent events and link OGM results with clinical information.

Regarding the workflow of the OGM technique, high genome-wide coverage (>500×) was achieved for all the samples without using additional Saphyr chips to repeat any sample runs. In our experience, it is very important to accurately quantify the amount of cells that are used in the DNA extraction and to follow the DNA quantification steps carefully after the extraction and labeling steps. The current limitations of OGM are mainly: (1) the requirement of fresh starting material, (2) the missing label sites at centromeres, (3) that exact breakpoints cannot be obtained at the sequence level, (4) the lowest limit of detection is slightly higher than for FISH, and (5) the throughput of a single instrument may not be sufficient for large laboratories (~3–6 cancer samples per 48 h). However, a single streamlined workflow for cytogenetic analysis could provide a significant reduction in costs and analysis time when compared with classical cytogenetic techniques [39].

## 5. Conclusions

Overall, our study demonstrated fully concordant results between SOC FISH and OGM in CLL patient samples. We determined that these aberrations are detectable within the range of 3–9% VAFs using OGM. In addition, the genome-wide analysis by OGM revealed additional chromosomal aberrations in the vast majority (78%) of the samples. These included many aberrations that have previously been reported in CLL, but also novel SVs of interest, including balanced translocations. Importantly, OGM enabled the detection of complex karyotypes, which are clinically relevant but undetectable by FISH. Overall, this study demonstrates the high potential of OGM to be used as a first-tier cytogenetic test for CLL and as a powerful tool for genome-wide SV analysis.

## Figures and Tables

**Table 1:** OGM results compared to SOC FISH results.

	FISH		OGM		
Sample ID	Material	Result (% of Positive Cells)	Material	Result (% Variant Allele Fraction)	Concordant with FISH
S1	BM	Trisomy 12 (68%)	BM	(12) × 3 (49%)	Yes
S2	Blood	Trisomy 12 (49%)	BM	(12) × 3 (45%)	Yes
S3	Blood	Trisomy 12 (60%)	BM	(12) × 3 (35%)	Yes
S4	Blood	Del(13q14.3) (87%)	BM	Del(13q14.2q14.3) (49,971,221–51,052,363) (89%) ^a^	Yes
S5	Blood	Del(11q22.3) (82%) Del(13q14.3) (89%)	BM	Del(11q22.1q23.3) (98,387,863–115,852,927) (43%) Del(13q14.13q14.3) (46,430,606–50,290,717) (57%)	Yes Yes
S6	Blood	Del(13q14.3) (96%)	BM	Del(13q14.2q14.3) (49,994,022–50,810,004) (94%) ^a^	Yes
S7	BM	Monosomy 12 (60%) Del(17p13.1) (70%)	BM	Del(12p13.1q12) (27,123,509–45,992,905) (31%) ^b^ Del(17p13.3p11.2) (66,653–21,732,588) (47%)	Yes Yes
S8	Blood	Del(11q22.3) (78%)	BM	Del(11q22.3q24.2) (107,890,357–126,343,687) (47%)	Yes
S9	BM	Del(13q14.3) (35%)	BM	Del(13q14.2q21.2) (47,507,135–60,435,220) (42%)	Yes
S10	Blood	Del(11q22.3) (18%)	BM	Del(11q22.1q23.3) (100,872,290–117,503,103) (11%)	Yes
S11	Blood	Negative	BM	Negative	Yes
S12	Blood	Negative	Blood	Negative	Yes
S13	Blood	Negative	BM	Negative	Yes
S14	Blood	Del(13q14.3) (13.5%) Del(17p13.1) (70%)	Blood	Del(13q14.q14.3) (49,282,594–51,131,202) (10%) Del(17p13.3p11.2) (66,653–22,079,438) (36%)	Yes Yes
S15	Blood	Negative	BM	Negative	Yes
S16	Blood	Negative	BM	Negative	Yes
S17	Blood	Del(13q14.3) (77%)	BM	Del(13q14.2q14.3) (49,952,447–50,937,582) (48%)	Yes
S18	Blood	Trisomy 12 (78%)	BM	(12) × 3 (47%)	Yes

^a^ Homozygous deletion based on VAF %. ^b^ OGM shows loss of the region that the FISH probes are targeting.

## Data Availability

The retrospective analysis made use of the data of the patients enrolled in The Finnish Hematology Registry and Clinical Biobank and was conducted according to the guidelines of the Declaration of Helsinki. The datasets generated during the current study are not publicly available but are available from the corresponding author on reasonable request.

## Supplementary Materials

The following supporting information can be downloaded at: https://www.mdpi.com/article/10.3390/cancers15041294/s1, Figure S1: An example of in silico dilution series to estimate lowest limit of detection with OGM; Figure S2: Identification of large rearrangements involving 13q14.3 region; Figure S3: The circos-plots view of three complex karyotypes identified by OGM; Figure S4: Identification of putative novel gene fusions; Table S1: List of known leukemia associated genes and their coordinates used in the analysis (hg38); Table S2: Estimation of the lowest limit of detection using in silico dilution series for representative aberrations; Table S3: Summary of technical quality metrics of the analyzed samples; Table S4: The number of rare SV calls, CNV segments and aneuploidy calls per sample; Table S5: Additional aberrations beyond the FISH targeted loci identified by OGM.
