# Supplementary material for: Optical Genome Mapping as an Alternative to FISH-Based Cytogenetic Assessment in Chronic Lymphocytic Leukemia
Source: Cancers (Basel). 2023 Feb 17;15(4):1294. doi: 10.3390/cancers15041294 (PMC9953986; doi:10.3390/cancers15041294)
Supplement: Supplementary file 1 [file cancers-15-01294-s001.zip › cancers-2193332-supplementary.pdf]

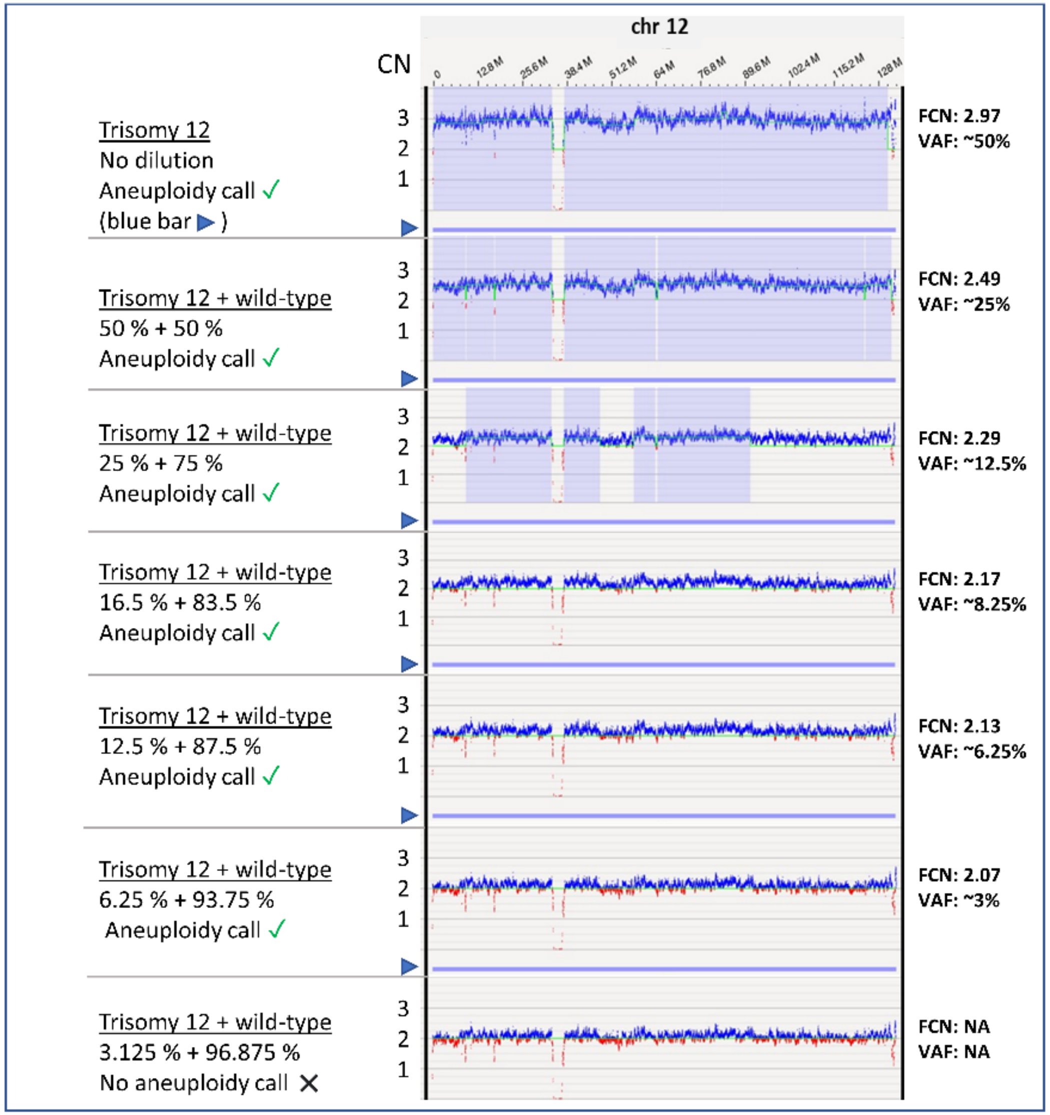

Figure S1. An example of in silico dilution series to estimate lowest limit of detection with OGM. The representative figure shows the process of in silico dilution of OGM data: molecule data of a sample with trisomy 12 is diluted with data from another CLL sample without trisomy 12 in stepwise manner until the trisomy 12 is undetectable with the aneuploidy calling tool. The CN value indicates the copy number, and the blue line below the CNV profile indicates an aneuploidy call made by the analysis software. Fractional copy numbers (FCNs) of the aneuploidy calls and the corresponding variant allele fractions (VAFs) are shown on the right side. The upper panel shows the undiluted sample, and each subsequent dilution step is shown below.

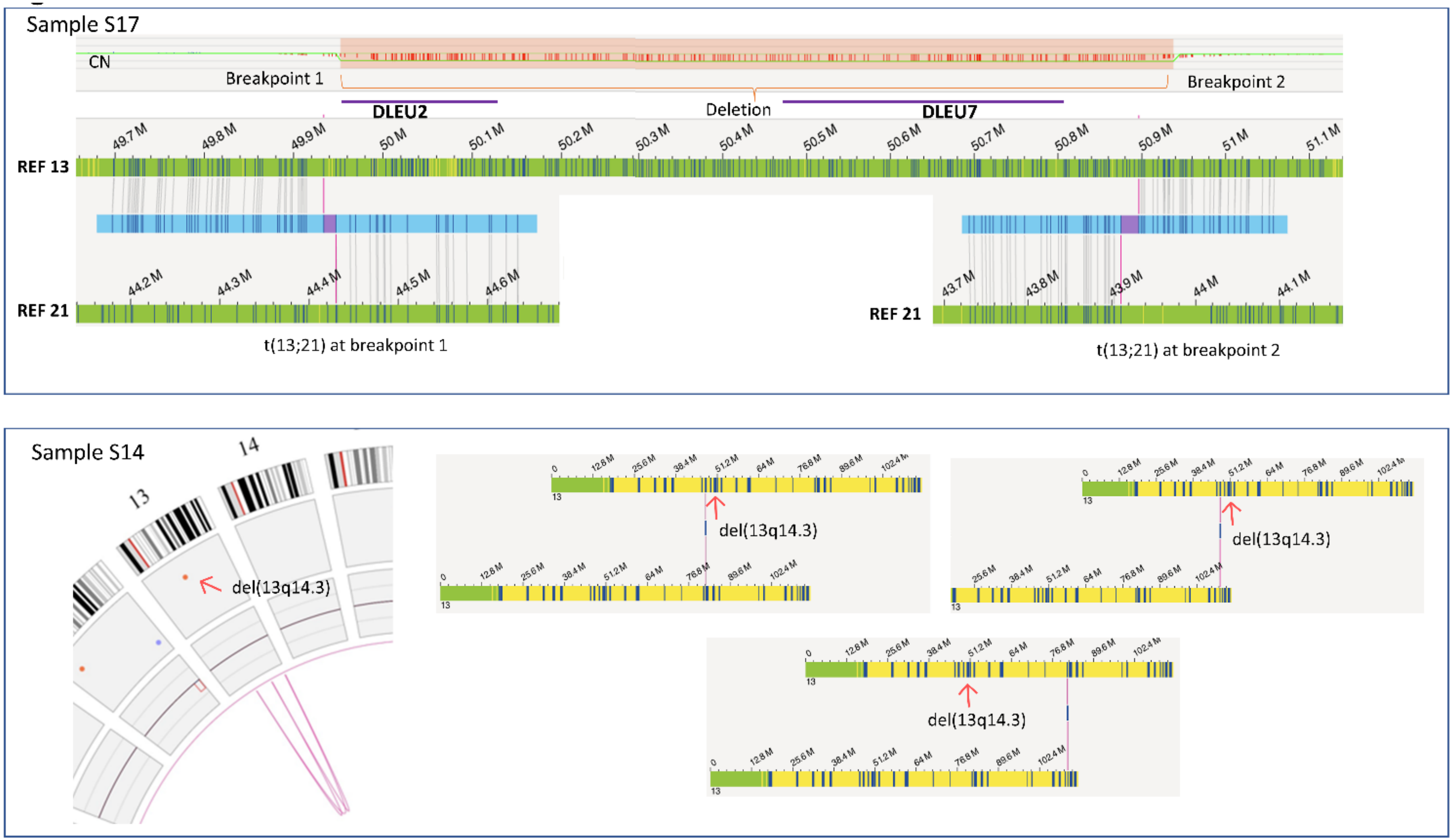

Figure S2. Identification of large rearrangements involving 13q14.3 region. Two cases showing large rearrangements involving the del(13q14.3) region. In sample S17, the OGM revealed two translocations t(13;21) where the breakpoints are on both sides of the deleted 13q14.3 region. In sample S14, the region with the deletion of 13q14.3 was involved in large intrachromosomal rearrangements of the long arm of chromosome 13.

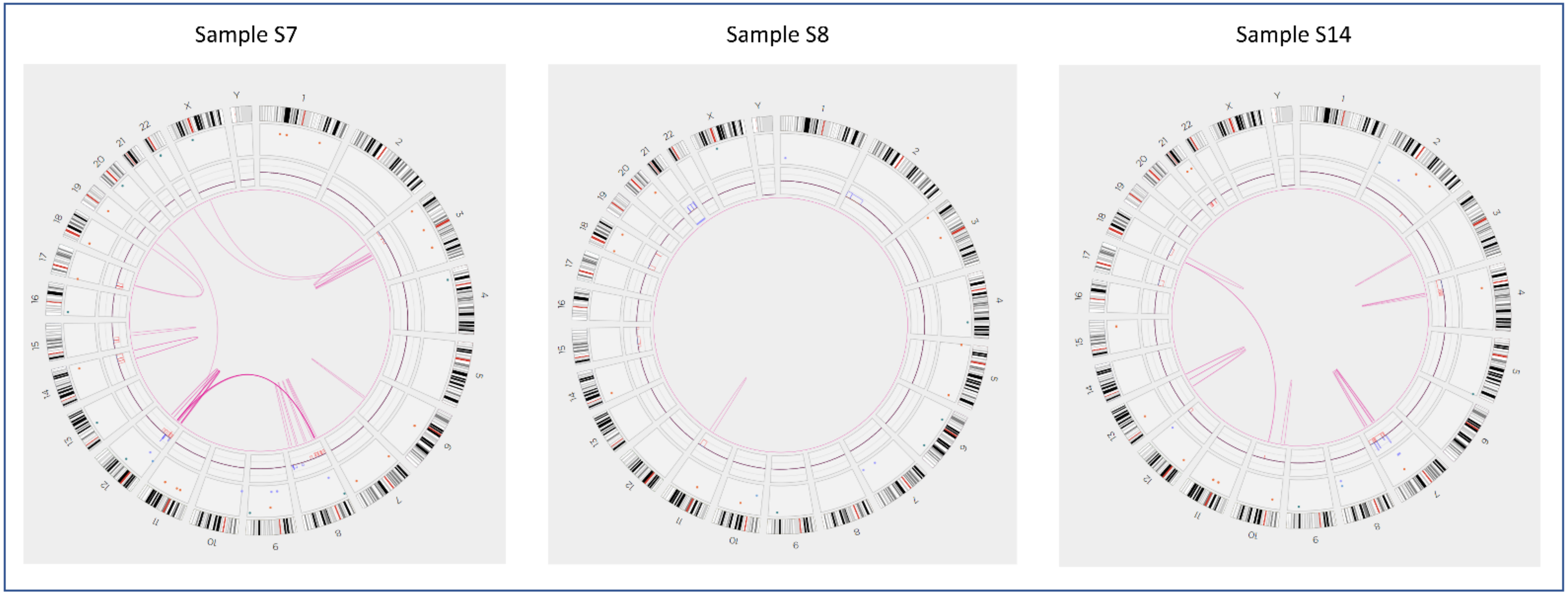

Figure S3. The circos-plots view of three complex karyotypes identified by OGM. Circos plot views of three samples in which OGM revealed complex karyotypes with 5 or more aberrations. Highly complex karyotypes (>5 aberrations) were identified in samples S7 and S14 harboring 17p deletion (*TP53*) while sample S8 (with *ATM* deletion) carried in total 5 large aberrations.

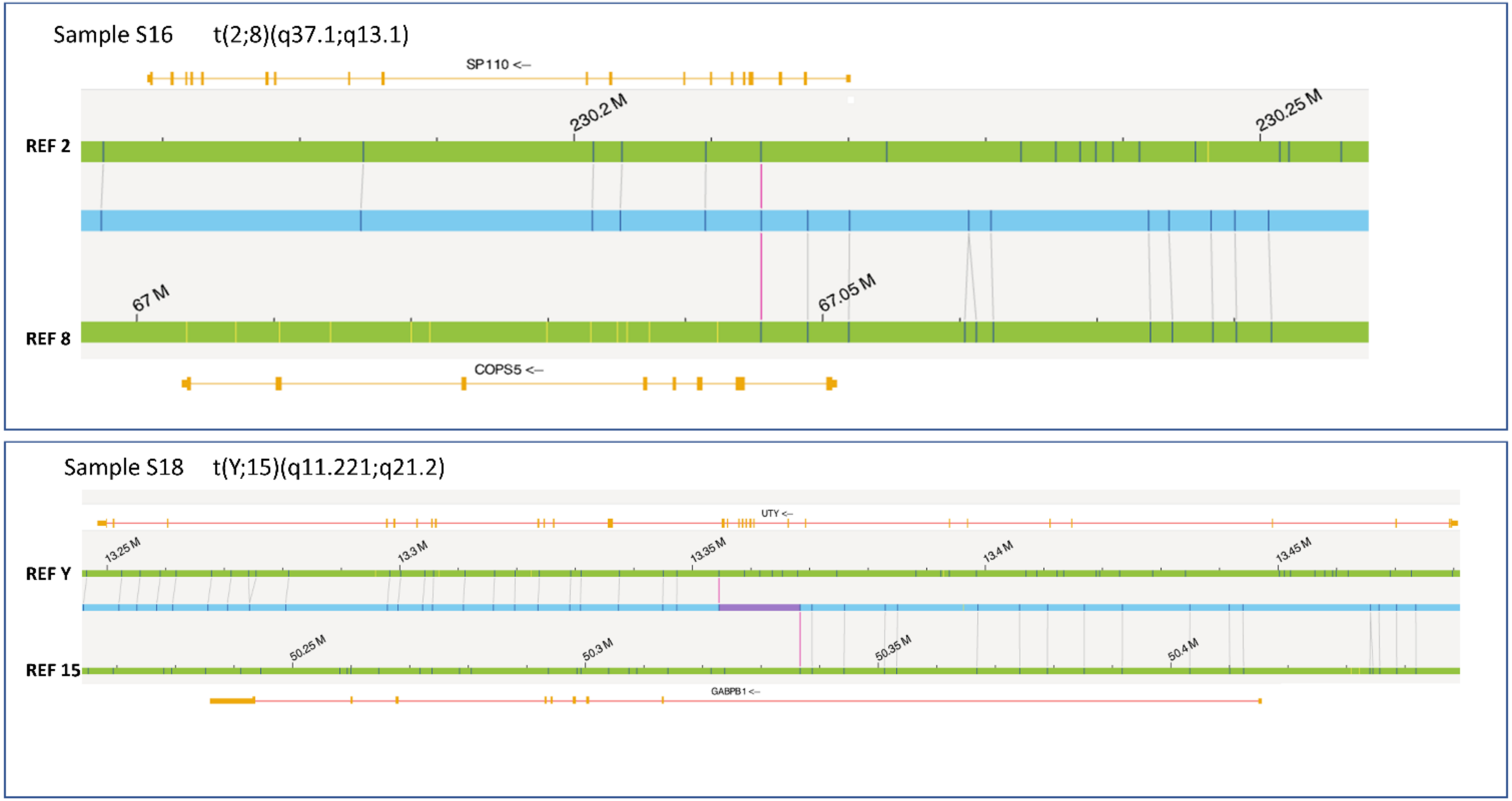

Figure S4. Identification of putative novel gene fusions. OGM revealed two balanced translocations leading to putative novel gene fusions. Sample S16 harbored t(2;8)(q37.1;q13.1), involving *SP110* and *COPS5*, as a sole aberration. Sample S18, with trisomy 12, carried a t(Y;15)(q11.221;q21.2) translocation involving *UTY* and *GABPB1*.
